# Enabling people with intellectual and other disabilities to make verbal requests using cardboard chips with mini objects or pictures and a smartphone

**DOI:** 10.3389/fresc.2023.1257493

**Published:** 2023-09-28

**Authors:** Giulio E. Lancioni, Nirbhay N. Singh, Mark F. O’Reilly, Jeff Sigafoos, Gloria Alberti, Oriana Troccoli, Isabella Orlando, Carlo Ricci

**Affiliations:** ^1^ Department of Neuroscience and Sense Organs, University of Bari, Bari, Italy; ^2^ Lega F. D’Oro Research Center, Osimo, Italy; ^3^ Department of Psychiatry and Health Behavior, Augusta University, Augusta, GA, United States; ^4^ College of Education, University of Texas at Austin, Austin, TX, United States; ^5^ School of Education, Victoria University of Wellington, Wellington, New Zealand; ^6^ Department of Psychology, Salesian Pontifical University of Rome, Rome, Italy

**Keywords:** verbal requests, intellectual disability, sensory impairment, motor impairment, technology, smartphone, cardboard chips

## Abstract

**Objective:**

This study aimed to help six participants with intellectual disability combined with sensory and motor impairments to make verbal requests through the use of a technology system involving cardboard chips and a smartphone.

**Method:**

The participants were divided into two groups of three based on whether they did or did not have visual skills. Each group was exposed to the intervention with the technology system according to a non-concurrent multiple baseline across participants design. During the 20 min intervention sessions, the participants were provided with a smartphone and nine cardboard chips each of which had a picture or object (i.e., a mini object replica or raised object contour) and several radio frequency identification tags attached to it. To make a request, the participants were to bring a cardboard chip in contact with the smartphone. This read the tags attached to the cardboard and verbalized the request related to that cardboard.

**Results:**

During the baseline (without cardboard chips and smartphone), the participants’ mean frequency of independent requests (all non-verbal requests) varied between zero and near 1.5 per session. During the intervention (with cardboard chips and smartphone), the participants’ mean frequency of independent requests (all verbal requests) varied between over 4.5 and about 10 per session.

**Conclusion:**

The results suggest that the system might be useful to help participants like the ones included in this study to make verbal requests with simple responses.

## Introduction

People with intellectual disabilities combined with sensory or sensory-motor impairments tend to have serious difficulties in critical areas of their daily life (e.g., functional occupation, access to leisure, and communication) (1–5). With regard to communication, a primary cause of their problems may be the absence or limited availability of verbal skills (6–11). Lack or minimal use of verbal skills can seriously curtail their opportunities of engaging in expressive communication, particularly their opportunities of making clear requests, with negative implications for their social interaction and possibility of accessing preferred environmental events (8, 9, 11–15).

To address this situation and minimize its negative impact, families and education and rehabilitation staff are typically advised to use non-verbal communication methods (16–18). The main non-verbal methods rely on manual signs, Picture Exchange Communication Systems (PECS) and Speech Generating Devices (SGDs) (19–23). All these methods have been widely employed and a vast literature is available that supports their applicability and beneficial effects (24–26). Notwithstanding the encouraging evidence available, one needs to examine the possible limitations/weaknesses of the single methods when considering their use with people with intellectual and other disabilities [e.g., intellectual disability combined with sensory and motor impairments (27, 28)].

For example, the PECS approach would not be suitable for participants whose condition also includes blindness (13, 29) given that blindness would preclude the identification and discrimination of the pictures involved in the communication exchanges (13). In an attempt to reduce the effects of this limitation, some studies have resorted to the use of three-dimensional symbols instead of pictures (13, 30, 31). Another possible weakness of the PECS method is that it may not work when the participant’s intended communication partner is not in the participant’s proximity and/or cannot be physically approached (e.g., because of the participant’s visual or motor impairments). This latter weakness is also characteristic of the manual signs method. In fact, any sign made when the intended communication partner is not present or is not watching the participant is practically destined to be ineffective.

SGDs are technically capable of overcoming the latter weakness of PECS and manual signs methods. Indeed, a SGD is capable of verbalizing the requests that a participant makes through simple responses such as touching specific pictorial images on a tablet screen. The verbalization of the requests is expected to alert the intended communication partners (even if they are not in the proximity of the participant) and eventually lead them to respond to such requests thus establishing an effective communication/interaction process (32–34). While apparently advantageous compared to the PECS and manual signs methods, SGDs may not always be suitable for people with multiple disabilities (35, 36). For example, the use of SGDs (with pictures representing the communication/request options) would not be realistic when the participant’s disabilities include blindness. SGDs might also be hardly usable with participants’ whose disabilities involve poor control of fine motor responses, that is, responses such as screen touching and stroking necessary for making requests on computer/tablet devices (29, 37–40).

An attempt to set up a technology system that would be able to verbalize the participants’ requests and could be applicable also with participants with blindness or poor vision and participants with poor fine motor control was reported by Ricci et al. (29). The system entailed a smartphone and a series of mini objects or cardboard chips with pictures/photos attached to them. The mini objects and chips were supplied with special frequency-code labels, which made them recognizable by the smartphone (i.e., via its Near Field Communication, NFC module). Bringing a mini object or a cardboard chip in contact with the back of the smartphone (i.e., the NFC module’s area) caused the smartphone to utter a verbal request for the corresponding event. Data showed that the five participants were successful in using the system, thus making a number of verbal requests.

The present study was aimed at extending the work carried out by Ricci et al. (29). In particular, it was to determine whether an updated version of the approach used by Ricci et al. (29) would be effective in helping six participants with combinations of intellectual disability and sensory and motor impairments to make verbal requests for preferred events. Being able to make clear (easily understood) verbal requests was thought to be an important achievement for the participants, that is, an achievement that would enhance their interaction/communication effectiveness, increase their access to relevant environmental events, and improve their personal and social outlook (29, 32, 35, 39). The basic components of the technology system were a smartphone and cardboard chips, each of which had a picture or object (i.e., a mini object replica or raised object contour) and several radio frequency identification (RFID) tags (41) attached to it. The presence of pictures or of different types of objects on the cardboard chips depended on the visual condition of the participants using the system and on their tactile discrimination skills. The presence of several tags [rather than a single one, as done by Ricci et al. (29)] on the cardboard chips was to facilitate/improve the identification of the chips by the smartphone independent of the accuracy with which the participants brought the chips into contact with the smartphone. The position of the smartphone and cardboard chips during the sessions differed across participants based on their general characteristics (e.g., visual and motor conditions). Of the six participants, three used the cardboard chips with pictures while the other three used the cardboard chips with objects.

## Method

### Participants

Table 1 lists the six participants through their pseudonyms and reports their chronological age, their sensory (visual and auditory) conditions, and their age equivalents as measured via the second edition of the Vineland Adaptive Behavior Scales (42, 43). The chronological age varied between 49 and 63 years. The sensory condition of the first three participants showed that two had a functional visual level (i.e., Thea and Rory) while the third (i.e., Evie) had poor residual vision, which allowed her to discriminate relatively large color pictures under optimal illumination. Two of these participants (i.e., Thea and Evie) also presented with deafness. Rory, who had functional hearing, received mechanical ventilation through a tracheostomy tube and was confined to a sedentary position. The sensory condition of the last three participants showed that they had total blindness. Two of them (i.e., Carson and Joel) also had deafness. Carson was moreover unable to walk, had arm spasticity, and spent his time in a wheelchair. All participants presented with limited/poor fine motor response skills. Specifically, they were unable to perform fine touching and stroking responses such as those that would be needed for selecting/activating stimuli on a computer or tablet screen.

**Table 1:** Participants’ chronological age, sensory conditions, and Vineland age equivalents for daily living skills (personal sub-domain) (DLSP), receptive communication (RC), and expressive communication (EC).

Participants (pseudonyms)	Chronological age (years)	Sensory conditions	Vineland age equivalents^a^^,^^b^		
			DLSP	RC	EC
Thea	63	Functional vision and deafness	5;6	4;8	2;1
Evie	61	Poor residual vision and deafness	5;3	4;8	2;3
Rory	59	Functional vision and functional hearing	2;1	4;8	3;4
Carson	53	Blindness and deafness	1;5	2;2	2;1
Joel	49	Blindness and deafness	3;11	2;2	1;7
Tristan	51	Blindness and functional hearing	3;1	3;8	2;1

^a^ The age equivalents are based on the Italian standardization of the Vineland scales (42).

^b^ The Vineland age equivalents are reported in years (number before the semicolon) and months (number after the semicolon).

Their Vineland age equivalents were between (a) 1 year and 5 months and 5 years and 6 months on daily living skills (personal subdomain), (b) 2 years and 2 months and 4 years and 8 months on receptive communication, and (c) 1 year and 7 months and 3 years and 4 months on expressive communication. Communication typically occurred through various gestures. Pictures or objects representing common activities/events were also used particularly by staff to inform the participants as to what was about to happen or to instruct them as to what they were expected to do. Tristan and Rory were the only ones who could understand a number of simple verbal expressions. None of the participants had received formal intelligence testing due to their complex condition. Estimates of their intellectual functioning available through the psychological services of the rehabilitation and care centers that they attended typically placed them within the moderate intellectual disability range.

The participants’ recruitment for the study was based on a number of conditions. First, all participants were known to lack effective expressive communication means. Indeed, they were apparently unable to make verbal requests. Moreover, their occasional gestures/signs could easily fail to attract the attention of staff unless staff were in their proximity and paid visual attention to them. Second, the participants were known to be interested in a number of events, which could include occupational or domestic activities (e.g., sorting clothes and watering plants), food items, music or videos, and forms of interaction with specific staff members (e.g., greeting them or going for a short walk with them). This interest in various events, which had been reported by staff and caregivers and confirmed by preliminary observations carried out within the participants’ daily contexts, did not seem to translate in the participants’ initiative to make requests for such events (perhaps because of their lack of effective request means; see above). Third, staff (a) considered it relevant to provide the participants with a simple technology that would allow them to make effective requests, and (b) thought that such technology could be made available during periods of the day when, although not in the participants’ immediate proximity, they would be able to respond to audible/clear requests.

### Ethical approval and informed consent

The use of the technology system planned in this study was considered to be a positive experience for the participants, as it allowed them to choose (request for) events and activities apparently interesting for them (see Participants). While their interest in events and activities available during the study could be taken to suggest their positive attitude toward the study (willingness/assent to be involved in the study), no evidence could be gathered about it, as they were unable to read and sign a consent form. This inability required their legal representatives to be involved in the consent process and to read and sign such consent on the participants’ behalf. The study complied with the 1964 Helsinki declaration and its later amendments and was approved by an institutional Ethics Committee.

### Setting, sessions, events/activities, cardboard chips, and research assistants

The study was carried out within the rehabilitation and care contexts that the participants attended. The baseline and intervention sessions were typically carried out within the participants’ occupational areas. An exception to this occurred for Rory who, given her physical condition, received part of her sessions in her bedroom. Sessions were 20 min long. This length was decided for three reasons. First, it was considered adequate to determine whether the participants would engage in requests that could easily alert the context (i.e., could be easily heard and understood) even when the research assistants or staff were not in the immediate proximity and/or were not paying visual attention. Second, it was considered to be a reasonable interval for the participants to express their requests and get those requests noticed and eventually satisfied. Third, it was hoped that such an interval could be viewed as acceptable (practically manageable within a daily schedule) by staff working in care and rehabilitation contexts.

Nine different events/activities were available at each intervention session for the participants to request. The events/activities, which were specific for each participant and involved some changes across sessions, could include: music, videos, shoulder and neck massage, food and drink items, telephone calls, short walks, small domestic activities (e.g., watering plants or putting away clothes) and occupational or assembly activities with different sets of material. Given their different nature, the events/activities (a) had different duration (e.g., from a few seconds to several minutes) and (b) required different types of responses from the context (e.g., simple delivery of a food or drink item, provision of a short massage, or assistance for a short walk).

The cardboard chips’ dimensions were 7 × 7 × 0.6 cm. The chips used for the first three participants were covered with the pictorial images of the events/activities represented. For example, an image could involve the photo of a person that could be contacted via video call, the photo of a singer, the representation of a video, the photo of the objects included in an activity, and the photo of a food or drink item. The cardboard chips available were based on the participants’ interests and sensory conditions. For example, chips with pictures of videos were not available for Evie (given her poor residual vision) while chips with images of singers/songs were available only for Rory (i.e., the only one with functional hearing). All images were discriminated and associated to their referents by the participants. The cardboard chips used for the last three participants contained mini object replicas or embossed objects (raised object contours) that represented the events/activities available for request. For example, a small bottle replica or embossed bottle shape could be used to represent a specific drink item, a small replica or flat shape of a dessert or yoghurt container could represent those items, a raised contour of a walking path could represent going for a walk, and specific object parts could represent activities involving the use of those objects. The mini replica and embossed objects were already discriminated by the participants and associated to the events/activities represented. The research assistants were three psychology graduates who had work experience with people with disabilities, were familiar with technology-aided interventions and data recording procedures, and had received preliminary staff coaching to familiarize with the participants and identify their requests within the daily context.

### Technology system

The technology system used during the intervention sessions included (a) a Samsung smartphone with Android operating system, which was fitted with the MacroDroid application, and (b) cardboard chips, which contained pictures or objects (i.e., mini object replicas or embossed objects) and RFID tags. Each cardboard chip was covered with multiple RFID tags so as to increase the chances that the smartphone’s NFC would readily recognize it. The MacroDroid application served to achieve two basic objectives, that is, to enable the smartphone to recognize/discriminate the different cardboard chips based on the tags attached to them, and to make the smartphone verbalize the request connected to each of those cardboard chips.

**Figure 1:**
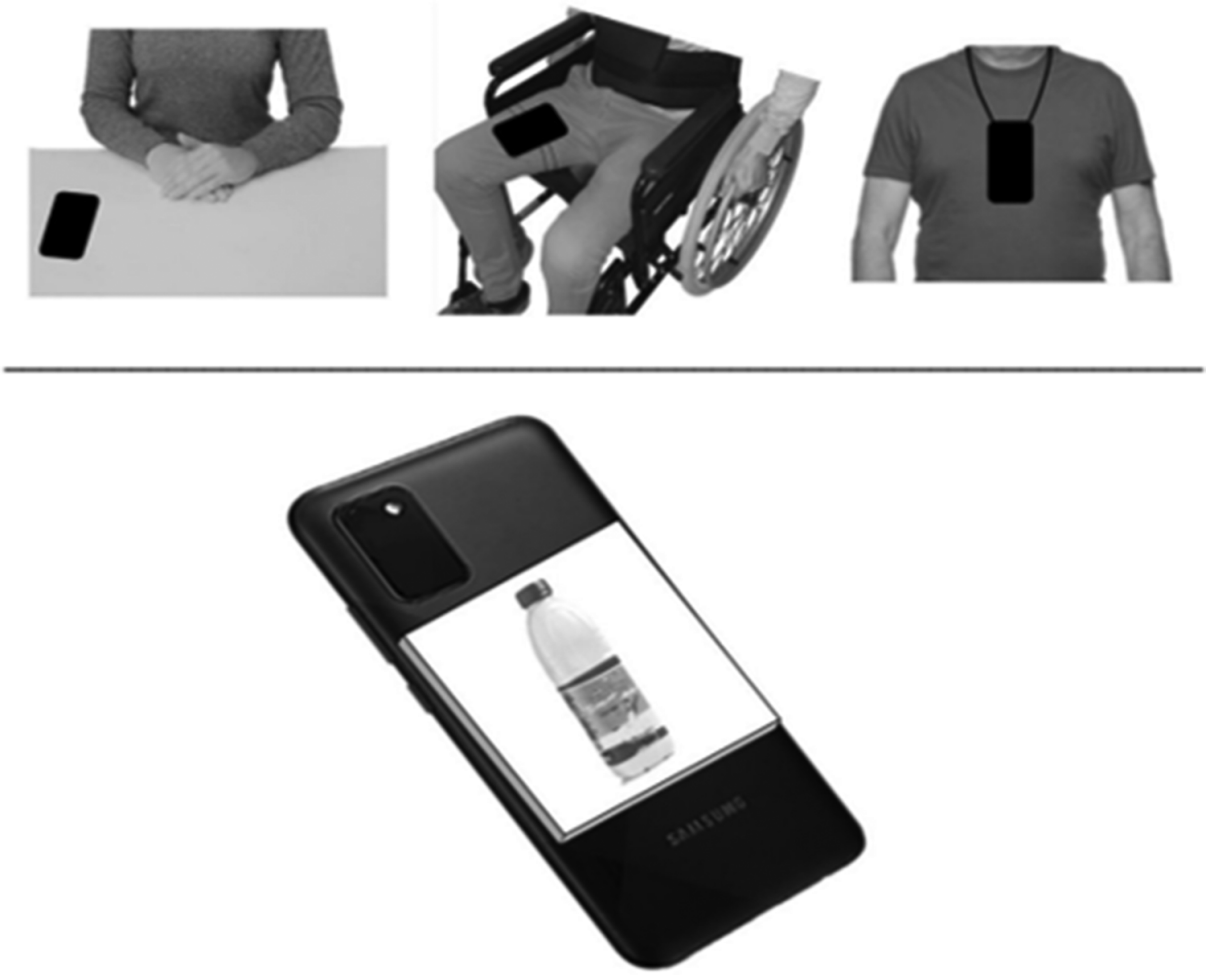
The upper section of the figure represents the position of the smartphone for different participants. The lower section of the figure represents the position of a cardboard chip on the smartphone as required for making a request.

At each intervention session, the participant sat at a desk (with the exception of Rory who stood in front of a desk), and was provided with the smartphone and nine cardboard chips. The smartphone was on the desk for the first three participants, tied to the right leg for Carson, and fixed at the chest for Joel and Tristan (see Figure 1, upper section). The smartphone screen was facing the desktop and the participants’ leg or chest, so all participants had direct/easy access to the smartphone’s NFC module, at the smartphone’s back, when making their requests (i.e., when bringing the cardboard chips into contact with the smartphone; see Figure 1, lower section). The cardboard chips were on the desk for the first three participants, on a bag tied to the wheelchair’s right armrest for Carson, in a belt bag for Joel, and in a little box for Tristan. The location of the smartphone and cardboard chips was adapted to the participants’ sensory-motor and response characteristics to facilitate their performance of request responses.

As soon as a cardboard chip was recognized by the smartphone’s NFC, the MacroDroid application ensured that the smartphone would produce a vibration (a feedback for the participant) and verbalize the request message associated with that cardboard chip. For practicality reasons, the verbalization was very short. It could entail a single word (i.e., the name of the item/activity requested) or two to four words including, for example, the name of the singer to listen to or of the person to call on the telephone. Following a request verbalization, the research assistant responded by satisfying such request (e.g., walked close to the participant and provided the food or drink item requested, the material necessary for the activity requested, or set up the telephone call with the person indicated in the request). Delivery and consumption of a food or drink item could be very fast and could also lead the participant to request for more of it (to make a new request for it). Some activities (e.g., occupational/assembly activities, watching videos, having a massage) could last several minutes and required different levels of involvement from the research assistant. This could vary from providing the activity material or starting a video on the smartphone to ensuring assistance during a walk or giving a massage.

### Measures and data recording

The measures recorded during the baseline and intervention sessions were (a) the requests the participants made independent of research assistants’ prompts (and whether those requests were satisfied), and (b) the instances of research assistants’ prompts to promote requests (see *Baseline* and *Intervention*). Research assistants were responsible for recording the measures. To increase the accuracy of recording and facilitate the computation of interrater agreement, the sessions were divided into 20 1 min intervals and requests and prompts were recorded within the intervals in which they occurred. Requests were quite easy to detect and record during the intervention (as they were characterized by a clear/audible verbal output). Yet, they could be less obvious during the baseline as participants could use subtle gesture/sign expressions as request means. To ensure that research assistants were able to recognize and record those means/requests, a period of coaching with staff had been arranged for them prior to the start of the study (See *Setting, sessions, events/activities, cardboard chips, and research assistants*).

Interrater agreement was checked in all baseline sessions and more than 20% of the intervention sessions through the involvement of a reliability observer (i.e., a second research assistant or a staff member) in data recording. The percentage of agreement (computed for each session by dividing the number of intervals in which the two raters reported the same frequency score for each of the measures by the total number of intervals and multiplying by 100%) ranged between 90% and 100%, with means exceeding 98%.

### Experimental conditions and data analysis

The first three participants (with visual skills) and the last three participants (with total blindness) formed two different groups, for each of which a non-concurrent multiple baseline across participants design was used (44, 45). In practice, the members of each group received different numbers of baseline sessions before the start of the intervention phase. During this phase, the participants were provided with the technology system. In order to guarantee a high level of procedural fidelity during the study [i.e., a high level of accuracy in the research assistants’ implementation of the baseline and intervention conditions (46)], two precautions were adopted. The first precaution consisted of providing the research assistants with the opportunity to practice their role before the beginning of the study (i.e., through two simulated baseline and intervention sessions). The second precaution consisted of providing the research assistants with supervision during the study. Supervision, which was ensured via a study coordinator who had free access to video-recordings of the sessions, consisted of giving the research assistants feedback and guidance to strengthen their performance accuracy. The end of the intervention was followed by a staff survey to identify staff opinion about the system and its overall potential.

The percentage of non-overlapping data method [PND (47)] was used to determine the effects of the intervention on the participants’ request behavior. This method allowed one to establish the percentage of intervention data points that indicated a larger frequency of requests than the highest baseline data point for each of the participants.

### Baseline

During the 5–9 baseline sessions, the participants sat at a desk or stood within a regular occupational room, and could have occasional interactions with staff. The research assistants presented the participants with prompts after intervals of 4–5 min had elapsed during which they had not produced any recognizable request. Prompts consisted of the research assistant presenting the participants with the gestures and/or material concerning two events/activities selected for the study (i.e., a food or drink item and objects to be assembled), asking them to choose/request the one they wanted, and satisfying the one they chose. Any choice/request connected to research assistant’s prompts was not counted as an independent request.

### Intervention

The intervention phase was preceded by five to eight introductory sessions in which the participants (a) familiarized with the technology system, (b) received research assistants’ visual, verbal and/or physical prompts to practice and consolidate request responses (i.e., to bring the cardboard chips in contact with the smartphone), and (c) had their requests satisfied via the research assistants’ delivery of the corresponding items, activity material, or physical input/support. At the start of each of the 61–85 regular intervention sessions, which followed the introductory sessions, the research assistants (a) provided the participants with the technology system, (b) waited for the participants to make requests (i.e., to bring cardboard chips in contact with the smartphone) and have the requests verbalized by the smartphone, and (c) ensured that those requests would be followed by the appropriate responses (i.e., delivery of the related events). For example, if the participants requested a food item, the research assistants provided that item (e.g., a biscuit or a few spoonsful of pudding). If the participants requested an activity (e.g., hanging out the laundry or making a phone call to a certain partner), the research assistants would accompany the person to the laundry area so the participants could carry out the activity or started a video call with that partner. If the participants requested an activity that entailed research assistants’ direct involvement (e.g., neck and shoulder massage or going for a walk), the research assistants provided the massage or accompanied the participants through the short walk. This could consist of pushing the participant’s wheelchair (i.e., Carson), or walking arm in arm through some of the immediate areas within the context and possibly greeting some staff available in those areas.

**Figure 2:**
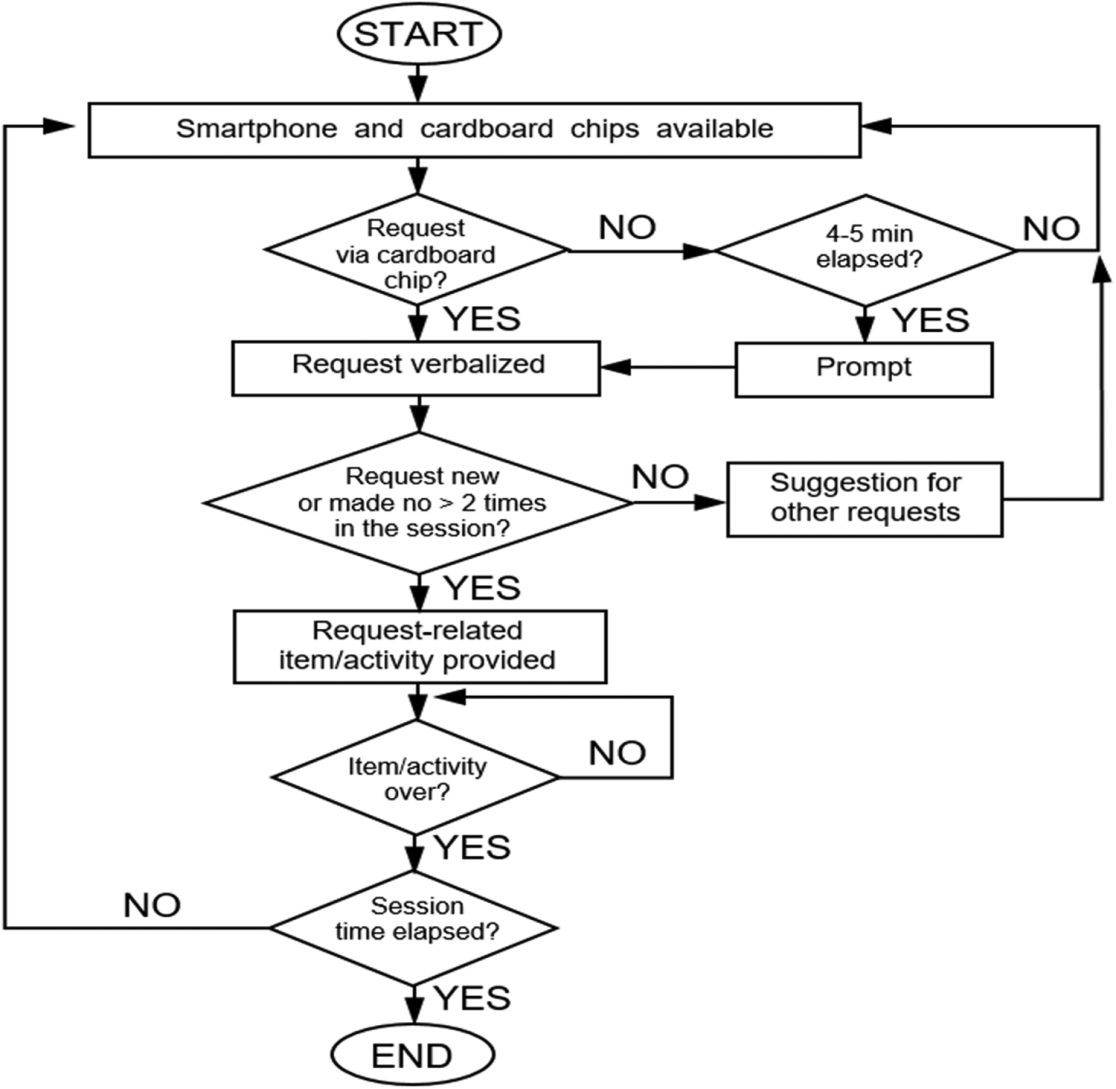
The flowchart summaries the intervention conditions.

Up to three requests for the same item or activity (e.g., a specific food) would be satisfied. Any additional request for the same item/activity was not recorded and not satisfied. The research assistants told the participants to choose something else and suggested two possible alternatives. The request for one of the alternatives suggested or any other alternative was considered to be an independent request and recorded as such. The research assistants could provide verbal, visual and/or physical prompts to promote participants’ requesting typically after intervals of 4–5 min had elapsed without the occurrence of an independent request. Any request that followed the occurrence of prompts was not counted and not included among the independent requests reported in the data. Figure 2 provides a summary of the conditions applying during intervention sessions.

The time needed for the research assistant to satisfy a request and the time the participants remained busy once the request was satisfied varied substantially in line with the different requests and the participants’ characteristics. For example, providing a food or drink item and consuming such an item would take a short amount of time (e.g., about 30 s). Setting up a video call with a partner could take little time to the research assistant and engage the participant for variable time periods (e.g., between 0.5 and 2 min). Providing the material for an assembly task could take little time to the research assistant and engage the participant for 2–5 min. Providing a massage or the guidance for a walk could take the research assistant 2–4 min and engage the participant for identical time periods.

### Staff survey

The survey was carried out with 30 staff members who were working in the rehabilitation and care contexts that the participants attended but had no connection with the participants. They were 27 females and 3 males whose age ranged from 25 to 54 (M = 39) years. The survey consisted of (a) showing single staff members or groups of up to four staff members a 3-min video with clips of the participants’ requests via the technology system and (b) asking them to provide their rating on four questions related to the video. The questions concerned the suitability of the system for the participants, its friendliness to the participants, its effectiveness in promoting independent and successful requesting, and its applicability during specific daily periods within regular rehabilitation and care contexts, respectively. The rating could vary from 1 (i.e., least positive value) to 5 (i.e., most positive value) (48, 49).

## Results

**Figure 3:**
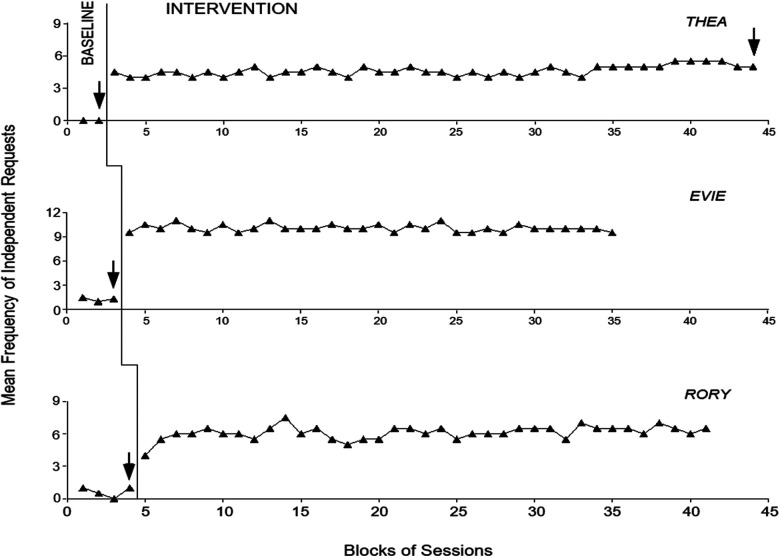
The three panels report the baseline and intervention data for Thea, Evie and Rory. Black triangles represent mean frequency of independent requests over blocks of two sessions. Blocks of three sessions are marked with arrows. The values on the ordinate axis differ across participants.

**Figure 4:**
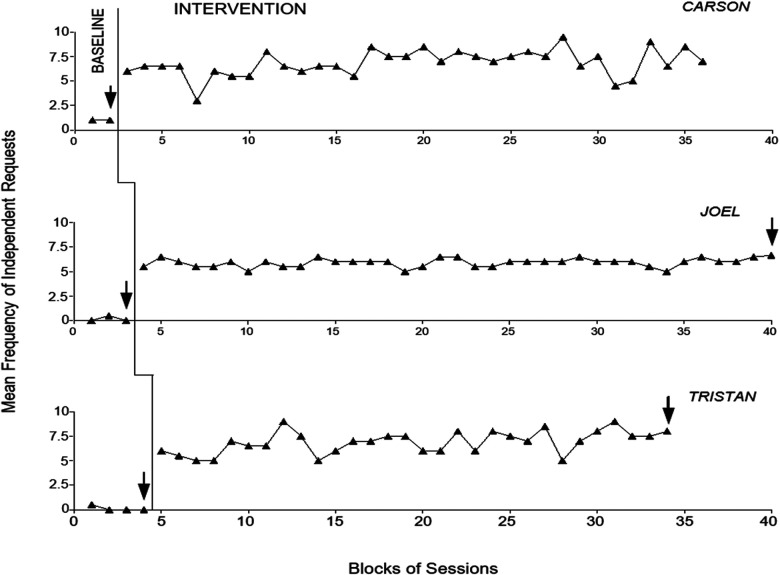
The three panels report the baseline and intervention data for Carson, Joel and Tristan. The data are plotted as in Figure 3.

Figures 3 and 4 report the baseline and intervention data for the first three participants and last three participants, respectively. The black triangles indicate the mean frequency of independent requests per session over blocks of sessions. The blocks, which are used to simplify the data display, include two and only occasionally (i.e., at the end of the phases) three sessions. The latter blocks are marked with an arrow. The figures do not report the introductory sessions used at the start of the intervention phase.

During the baseline phase, the participants’ mean frequency of independent requests (all non-verbal requests) ranged between zero and near 1.5 per session. Staff mostly failed to notice and respond to the requests, but could have occasional/brief interactions with the participants independent of requests. During the intervention phase (i.e., with the use of the system), all participants were able to make multiple requests. Their mean frequency of independent requests (all verbal requests) varied between over 4.5 (Thea) and about 10 (Evie) per session. The frequencies were largely related to the specific events requested during the sessions and to the length of those events for the different participants. The events mainly involved (a) videos and photograph collections, occupational activities, and walks (i.e., with meeting of specific staff members) (Thea), (b) occupational/domestic activities and food and drink items (Evie), (c) videos, songs, and video calls (Rory), (d) food and drink items and walks (Carson), (e) occupational and recreational activities and walks (Joel), and (f) occupational activities, food or drink items, songs, massage and walks (Tristan).

Research assistant’s prompts, which occurred regularly during baseline, were (virtually) absent and/or limited to the initial sessions during the intervention. The only exception was Carson who continued to receive prompts (i.e., a mean of about one prompt per session) throughout the intervention phase. In fact, he tended to make requests for the first part of the session and then enter a period of passivity. Yet, he would restore independent requesting after research assistant’s prompts. The intervention data points (i.e., frequency of independent requests) regularly exceeded the highest baseline data points for each of the participants, thus the PND index was always 1 confirming the strong impact of the intervention (47).

The staff survey provided mean scores varying from 4.6 to 4.2 for the four questions presented. It may be noted that a score of 4 represented a fairly positive answer (opinion) about the question presented. The first three questions concerned the suitability, friendliness, and effectiveness of the system (i.e., its appropriateness and relevance for the participants). The last question concerned the usability of the system during specific daily periods within regular rehabilitation and care contexts.

## Discussion

The results suggest that the technology system assessed in this study was helpful in supporting participants with intellectual, sensory, and motor disabilities to make verbal requests through simple responses suitable to their general conditions. These results (a) corroborate and expand the early evidence presented by Ricci et al. (29) and (b) indicate that the technology system employed might serve as an alternative to conventional SGDs for people who could not realistically use such devices due to their sensory impairments and their poor fine motor control (29, 39, 40). In light of the above, a few considerations may be in order.

First, giving people with extensive multiple disabilities the opportunity to make verbal requests through a relatively simple and friendly technology system may be considered a relevant objective to pursue for home and rehabilitation and care contexts. In fact, the use of verbal requests may be an effective way for these people to reach a communication partner (e.g., a staff member or caregiver) even when the partner is not in the immediate area and is not paying visual attention to them (19, 20, 34). The possibility of reaching their communication partners and eventually having their requests satisfied would certainly bear positive effects on their social interaction and general status [i.e., it would reduce their sense of isolation, frustration and failure, increase their access to relevant environmental events, and conceivably improve their level of satisfaction and quality of life (50, 51)].

Second, while a number of SGDs are available to support verbal requests, the use of those devices with people like the participants of this study may not be easy or possible (29, 38, 40). The reason is that most of these devices rely on visual symbols displayed on an electronic screen (e.g., tablet or iPad’s screen) and require that the participants make their requests by selecting the symbol and by touching/stroking the device’s screen area where that symbol is displayed (6, 52). The use of visual symbols on a screen would have been impossible at least for three of the participants included in this study (i.e., the three with blindness). Producing a fine touching/stroking response on a device’s specific screen area would have been almost certainly impossible for all six participants.

Third, the technology system used in this study can be considered easily accessible and largely affordable (53–57). The three basic components of the system (i.e., smartphone, RFID tags, and MacroDroid application) are commercially available and can be purchased for a modest sum. Indeed, a simple/basic smartphone with Android operating system may cost slightly above US$ 200, each tag costs about US$ 0.25, and the MacroDroid may be acquired for less than US$ 10. The cardboard chips, photos, and mini object replicas or embossed objects can easily be developed and produced by staff or caregivers within the daily contexts in which they operate.

Fourth, a range of nine request options within any single session may be considered fairly limited. One way to expand such a range might involve the use of extra cardboard chips. A more practical and realistic way to address the issue may consist of using the same number of cardboard chips, but ensuring that each of them produces a request for two similar events (e.g., two similar food items or occupational activities). The communication partner (e.g., research assistant, staff member or caregiver) responding to the request could present the participant with the two events and let the participant choose between them.

Fifth, the staff survey seemed to indicate that personnel familiar with people with multiple disabilities considered the system quite suitable for these people, friendly to them, and effective in supporting their requests. The same personnel also seemed to believe that the system could be used during specific periods of the day within regular rehabilitation and care contexts. This last point might be taken as encouraging about the possibility of adopting the system for daily use, thus allowing participants to have a more active role and eventually a more pleasant interaction with staff and caregivers.

### Limitations and future research

The main limitations of the study are the small number of participants, the lack of assessment of the participants’ satisfaction with the use of the system, and the absence of maintenance and generalization data. The first limitation calls for new (direct and systematic replication) studies to determine whether the data reported are robust and consistent across participants and thus whether it is possible to make general statements about the potential of the system (58–61). As to the second limitation, two viewpoints are possible. One viewpoint might take the participants’ consistent use of the system across sessions and the fact that the system allowed them to access typically positive events as two important elements for suggesting that the participants were most probably satisfied with the availability of the system. A second viewpoint might stress that a formal assessment of participants’ satisfaction is necessary, notwithstanding the reasonableness of the aforementioned suggestion. The formal assessment could be carried out by (a) recording the participants’ indices of happiness during the intervention sessions with the system and outside of those sessions, and/or (b) letting the participants choose between those sessions and other daily occupational situations (1, 10, 62, 63).

To address the third limitation of this study (lack of maintenance and generalization assessment), one would expect future research to extend the use of the system over larger numbers of sessions as well as across different contexts, staff members and caregivers. While one has to wait for new data to make general statements, it might be reasonable to anticipate that those data would tend to be positive if (a) the requests continue to be responded to (satisfied), and (b) the events provided in relation to the requests are sufficiently motivating (59, 64).

The use of a non-concurrent multiple baseline across participants design with each of the two groups of participants might be (a) viewed as a relatively weak choice when compared to the use of an ABAB (withdrawal) design for the single participants (45, 65), and thus (b) pointed out as another potential limitation of the study. In contrast with such a view, the choice was considered appropriate from a methodological and ethical standpoint. Given that none of the participants could be expected to learn to make clear/audible requests without the support of the technology, a second baseline would have been methodologically unnecessary and ethically censurable (65).

In conclusion, the results have shown that the technology system was effective in helping participants with intellectual, sensory or sensory-motor disabilities and reduced control of their fine hand movements to make verbal requests through the use of cardboard chips with pictures or mini objects. Although encouraging, these results do not allow one to make general statements about the potential and usability of the system given the aforementioned limitations of the study. New research is needed to amend those limitations and possibly upgrade the system to make it more easily applicable across individuals and settings.

## Data Availability

The raw data supporting the conclusions of this article will be made available by the authors, without undue reservation.
